# Drought analysis with different indices for the Asi Basin (Turkey)

**DOI:** 10.1038/s41598-020-77827-z

**Published:** 2020-11-26

**Authors:** Mehmet Dikici

**Affiliations:** Department of Civil Engineering, Alanya Alaaddin Keykubat University, Alanya, 07945 Turkey

**Keywords:** Hydrology, Civil engineering

## Abstract

Today, within the scope of planning, development and management of water resources affected adversely by climate change, the issue of minimization of the adverse effects of drought has become very important. In this study, drought risk analyses were performed using meteorological, hydrogeological and hydrological data of the Asi basin and as a result of the determination of different indices and indicators available in the literature. First, the missing data was completed by regional analyses. The DI (Deciles Index), SPI (Standardized Precipitation Index), SPEI (Standardized Precipitation Evapotranspiration Index) and SRI (Standardized Runoff Index) indices were described. Drought severity and magnitude were found according to these indices. Based on 1, 3, 6, 9, 12, 48-month recurrence intervals, analyses were made. Classification of droughts and their threshold values were determined. For some places, drought incidence rates were presented according to each index. The indices were compared, the correlation between them was examined and a common conclusion was reached. The drought severities, which has a precipitation area of 7800 m^2^, were evaluated for certain recurrence intervals. For this purpose, based on meteorological, hydrological and hydrogeological data for the years between 1970 and 2016, DI, SPI, SPEI, and SRI indices were analyzed and compared.

## Introduction

Today, within the scope of planning, development, and management of water resources adversely affected by climate change, the issue of mitigating the expected effects of drought has become very important. Climate change is increasing the pressure on water bodies. From floods and droughts to ocean acidification and rising sea levels, the impacts of climate change on water are expected to intensify in the years ahead. Cities and regions are already adapting, using more sustainable, nature-based solutions to lessen the impact of floods and using water in smarter, more sustainable ways to enable us to live with droughts^1^. Although the scientific world examines how the oceans and seas will be affected by global warming, which species will change their habitats, which species will disappear, how biodiversity will become, but it cannot make a full estimate because nature consists of a multi-component system. International climate change studies (IPCC) state that the sea level has risen globally 10 to 20 cm in the past hundred years, and this is predominantly due to global warming, and this century will increase 40–60 cm. Mediterranean; The ecological—oceanographic changes in the Atlantic Ocean and the Atlantic Ocean connected to the Strait of Gibraltar directly affect the Mediterranean. On the other hand, the Mediterranean; It is also open to changes in the Red Sea and the Indian Ocean^2^.

Many researchers discussed the drought situation combining with weather/climate conditions, such as severe weather occurrence frequency^3–8^.

The Mediterranean is one of the most important and vulnerable climate change “hot spots” and responds quickly to atmospheric events^9,10^. The region is known to affect human and natural systems such as water management, human health, plant/marine diversity, agriculture and socio-economic efficiency^11,12^. Also, the Euro-Mediterranean region often experienced extreme climatic and weather events, such as the hottest summer between 2003 and 2010^13,14^. The magnitude and frequency of extreme temperature events on land and sea tend to increase in recent years and are expected to increase in the future^15–19^.

Drought is defined as a natural disaster that occurs when precipitation is significantly less than its normal time. There are different types of drought that can occur at any time and anywhere. In case of lack of precipitation, meteorological drought is defined as deficiency in surface or groundwater hydrological drought. Agricultural drought, on the other hand, is expressed by the fact that precipitation, surface and groundwater deficiency are restricted by agricultural productivity. The most basic way to monitor drought is drought indices. Thus, it is possible to determine the duration and severity of the drought quantitatively. For example, in the eastern Mediterranean region adjacent to the Asi basin, there are increases in natural disasters due to extreme increases (such as excessive precipitation, extreme temperatures, storms and hoses, drought).

It is frequently used in the literature to determine drought, especially in the presence of precipitation, flow and evaporation as meteorological data^20–24^.

Managing the increased drought risk and adapting to this risk can only be achieved through the development of sustainable and effective drought risk-management strategies that adopt holistic approaches. Drought management is a part of the disaster management^25^. Drought risk management is the concept and study of preventing and mitigating the negative consequences and potential disaster impacts of drought hazard through activities and measures aimed at prevention, harm reduction, and preparedness^26^. Drought risk management constitutes an important part of the water-resource management policies and strategies. National drought policies have an important role in managing drought risk^27^. Research related to the future impact of climate change on the environment, especially on water resources, also sheds light on the measures that should be taken^28^. In order to mitigate the drought-related effects, it is necessary to prepare drought management plans by depending on the Country legislation and taking into account the specific drought characteristics and effects of the basin^29^. It is very important that these plans are prepared as part of the basin management plan in order for them to form integrity. In addition, the participation of all stakeholders, affected sectors, decision-makers and experts in the creation of plans contributes greatly to the success of drought management plans. Knowing the characteristics of the river basin, examination of historical drought events happened in the basin, evaluation of the risk that may occur, determination of the indicators and threshold values for drought analysis, creation of measurement program to reduce the impacts of the drought, and establishment of the early warning system and organization structure are among the elements of the drought management plan^30^. The drought risk management includes evaluation of the hazard, exposure and impact, the early warning system that includes affectability, drought observation and prediction, preparedness and harm reduction stages^25^. Early warning systems, one of the most important elements of drought management plans, are used within the framework of two goals, drought monitoring and drought prediction. Drought early warning systems typically aim to monitor, evaluate and present information related to climate, hydrological features, water supply conditions and trends. In order to take action within a drought risk management plan to mitigate any potential impacts, it is also aimed to provide information early before or during the onset of a drought^31^. Since drought is a slow-starting and progressive hydrological event, monitoring and analysis of it are of great importance.

Edwards^31^ analyzed 1221 stations in the United States in terms of drought and humidity by using the SPI method. In their study conducted in India, Patel et al.^32^ evaluated the drought index to determine temporal and positional drought risk by using long-term monthly precipitation series and by calculating SPI for 3, 6, 9, 12, and 24-month time periods. Same studies were conducted by Chen et al.^33^ in Taiwan and by Zhang et al.^34^ in China. Hong et al.^35^, Arabzadeh et al.^36^, Gumus and Algin^37^and Dikici^38^ conducted drought analysis studies by using runoff data. SPI index is also applied in other studies focusing on regional drought^39,40^. On the other hand, other studies examined the cause of long-term change in regional precipitation^41–44^. These studies also indicate the importance of the ocean and atmosphere forcings on the variation in regional precipitation. Whether or not there was a drought trend for the Asi Basin was previously studied by a trend analysis using meteorological and hydrological data^45^.

In this study, whether there was a drought trend for the Asi basin was addressed with the help of indices. For this purpose, based on meteorological, hydrological and hydrogeological data for the years between 1970 and 2016, DI, SPI, SPEI, and SRI indices were analyzed.

## Results

The results were evaluated under 3 headings: drought severity analyses, drought risk analyses and determination of the arid periods for 5, 10 and 50 years. Finally, they were obtained drought risk maps and drought recurrence-severity maps according to results.

### Drought severity analyses

#### Deciles index (DI)

**Figure 1:**
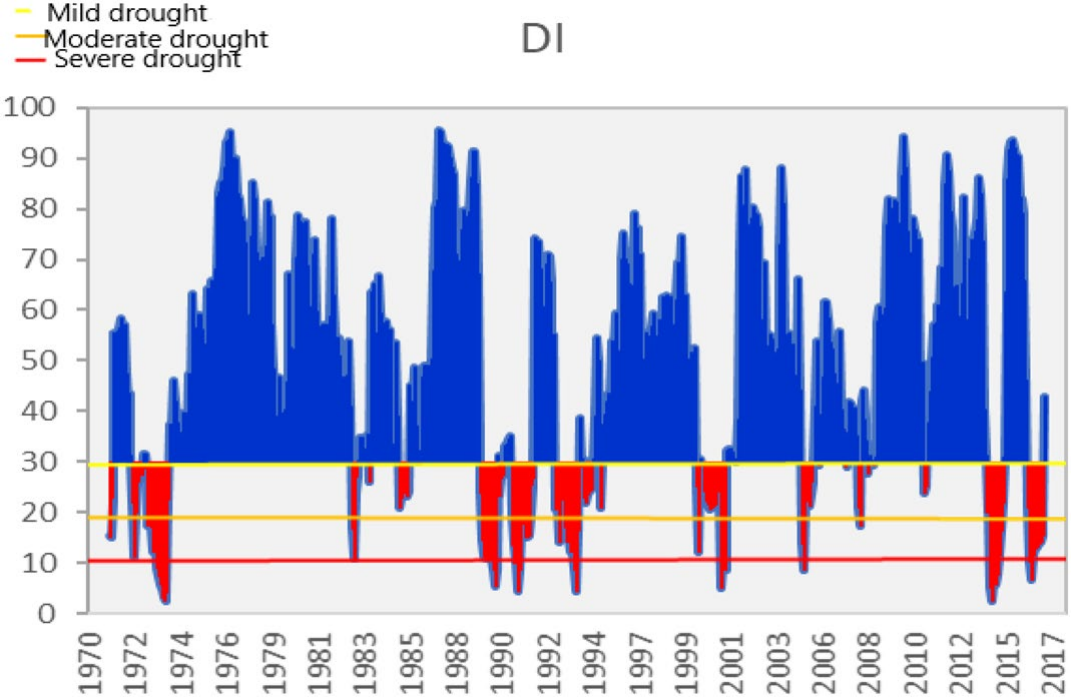
Asi Basin monthly DI severity-time series.

Monthly values for the Deciles Index (DI) were calculated for stations in the basin. Later, these values were made areal in a way to cover the whole of the Asi Basin by taking the weighted averages. The values calculated for the Deciles Index are used in calculations and are given in Fig. 1 as a sample. In the series, whereas the blue periods represent normal and above (humid) periods, the red periods indicate arid periods.

#### Standardized precipitation index (SPI)

1, 3, 6, 9, 12, 18, 24 and 48-month values of the Standardized Precipitation Index (SPI) were calculated for stations in the basin. Then, these values were made areal in a way to cover the whole of the Asi Basin by taking the weighted averages. SPI drought-severity time series of the Asi basin are used in calculations.

#### Standardized precipitation evapotranspiration index (SPEI)

The Standardized Precipitation Evapotranspiration Index (SPEI) was calculated in different periods (1, 3, 6, 9, 12, 18, 24 and 48-month) and the calculated SPEI values for the basin is presented in the time series below. The values for the basin were found by taking the weighted averages of SPEI values calculated on the basis of stations in the basin in the way to cover the whole of the Asi Basin and making them areal. SPEI drought-severity time series of the Asi basin are used in calculations.

#### Standardized runoff index (SRI)

In the context of the conducted study, the Standardized Runoff Index (SRI) was calculated for different periods (1, 3, 6, 9, 12, 18, 24, and 48-month). In order to make SRI time series consistent and comparable with other indices calculated in the scope of this study, data covering the same years were used. 1, 3, 6, 9, 12, 18, 24 and 48-month SRI severity time series for the basin are used in calculations.

### Drought risk analyses

**Figure 2:**
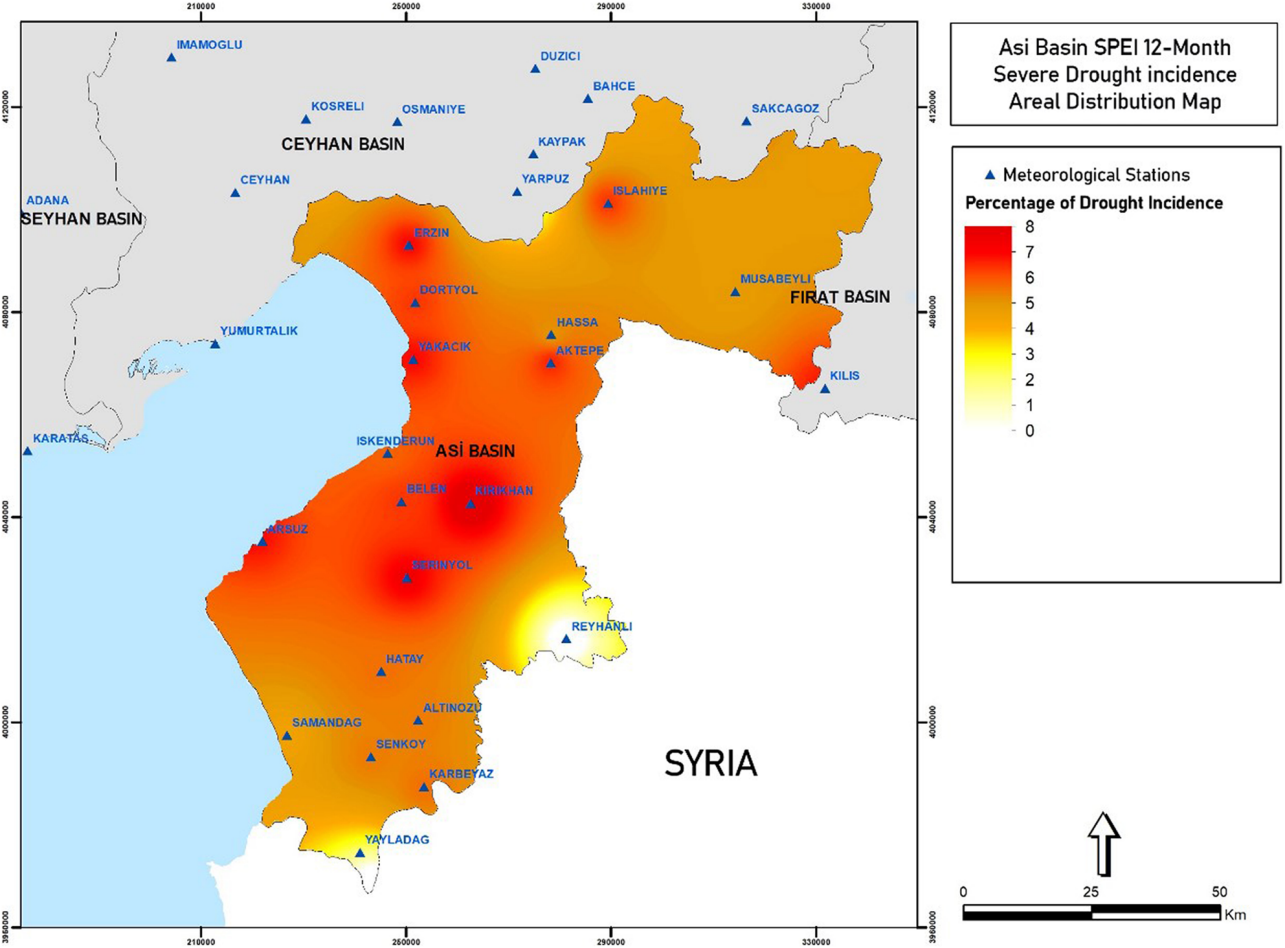
Asi Basin SPEI 12-month severe drought percentage (Global Mapper v19 and http://www.bluemarblegeo.com).

In this section, it is aimed to present drought risk analyses created using the calculated meteorological and hydrological indices. In order for them to represent the meteorological drought examined in this study, the values of the DI, SPI, and SPEI meteorological indices were analyzed, and in order for the past drought periods to be represented consistently, risk analyses of 12-month index values were conducted and given in Fig. 2 as a sample. It was generated via Global Mapper program.

#### Deciles index (DI)

**Figure 3:**
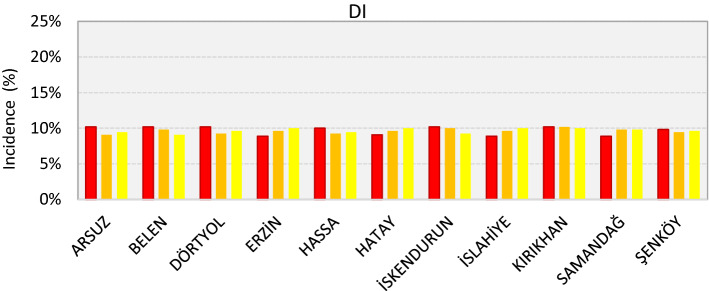
DI drought incidence rates of the stations in the Asi Basin.

Drought incidence rates were calculated according to the Deciles Index (DI) classification. The 12-month DI values of stations within the basin are presented in the chart below according to their incidence. Drought incidence rates according to the DI drought classifications of stations within the Asi Basin are used in calculations are given in Fig. 3.

According to the performed evaluation, it was identified that the highest incidence rate of mild drought was in Erzin, Hatay, Islahiye and Kırıkhan with 10.0%, and the lowest was in Belen with 9.1%. The moderate drought incidence rate was highest in Kırıkhan with 10.2% and lowest in Arsuz with 9.1%. The highest incidence rate of severe drought was observed in Arsuz, Belen, Dörtyol, Iskenderun, and Kırıkhan with 10.2%, and the lowest was seen in Erzin, Islahiye and Samandağ with 8.9%. According to the averages of the stations within the basin, it was calculated that the severe drought incidence rate in the basin was 9.7%, the moderate drought incidence rate was 9.6% and the mild drought incidence rate was 9.7%.

#### Standardized precipitation index (SPI)

Drought incidence rates were calculated according to the Standardized Precipitation Index (SPI) classification. The 12-month SPI values for stations within the basin are presented in the below according to their incidence frequencies. Drought incidence rates according to SPI-12 drought classifications of stations within the basin are used in calculations.

According to the performed evaluation, it was observed that the highest incidence rate of mild drought was in Erzin with 17.0%, and the lowest was in Arsuz with 8.5%. The moderate drought incidence rate was highest in Arsuz with 12.9% and lowest in Şenköy with 6.6%. The highest incidence rate of severe drought was observed in Dörtyol with 8.7%, and the lowest was seen in Kırıkhan with 6.1%. According to the averages of the stations within the basin, it was calculated that the severe drought incidence rate in the basin was 7.3%, the moderate drought incidence rate was 8.8% and the mild drought incidence rate was 13.9%.

#### Standardized precipitation evapotranspiration index (SPEI)

Drought incidence rates were calculated according to the Standardized Precipitation Evapotranspiration Index (SPEI) classification. The 12-month SPEI values of stations within the basin are presented in the below according to their incidence frequencies. Drought incidence rates according to SPEI-12 drought classifications of stations within the basin are used in calculations.

According to the performed evaluation, it was observed that the highest incidence rate of mild drought was in Belen with 17.9%, and the lowest was in Dörtyol with 6.3%. The moderate drought incidence rate was highest in Arsuz with 14.2% and lowest in Dörtyol with 6.3%. The highest incidence rate of severe drought was observed in Kırıkhan with 7.9%, and the lowest was seen in Samandağ with 4.4%. According to the averages of the stations within the basin, it was calculated that the severe drought incidence rate in the basin was 6.2%, the moderate drought incidence rate was 9.3% and the mild drought incidence rate was 13.6%.

### Determination of arid periods

The drought indices calculated for the determination of arid times of past periods in the Asi Basin were examined in detail. By the study conducted in this section, it was aimed to determine common periods between indices. The method used is described below:

- For the determination of the arid periods, 25 drought indices selected for the comparison were sorted monthly for the time period (1970–2016) examined under this study by bringing them to the same time period.
- By taking the areal weighted average of each index for the basin and using its own parametric drought classification, it was calculated whether the overall basin was above normal arid or not. At this level, only whether drought occurred was determined and the severity of the drought in itself was not studied. Comparative monthly time charts for the indices were prepared for the years 1970–1985, for the years 1986–2001, and for the years 2002–2016. The color scale used in the charts was determined based on the common classification determined for drought indices. According to this, while the white color refers to normal and above (moist) status, the yellow color is mild drought, the orange color is moderate drought, and the red color is severe drought indicators. In the context of this study, it was aimed to examine the periods that indicated common drought in the chart by determining them with comparisons between indices. In this context, for the Asi Basin, arid periods of various lengths were identified in the years 1973–1974, 1989–1991, 1993–1994, 2000–2001, 2004–2005, 2014 and 2016.

#### Maps of drought severity and magnitude

**Figure 4:**
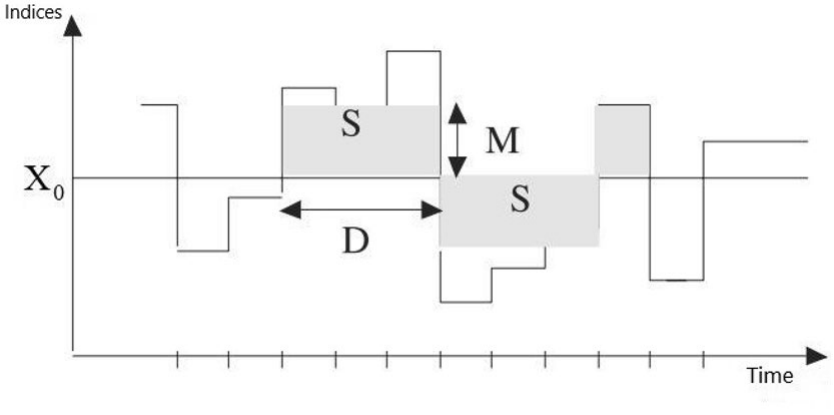
Parameters helping the definition of drought: S, M, D.

The drought severity (S), magnitude (M) and duration (D) values, which are criteria that allow a wide range of drought definitions, were defined by the help of Fig. 4.

Accordingly, the threshold value denoted by X_0_ is the value of zero, which is the arid/humid threshold of the indices under the project. The drought severity parameter, expressed by S, is obtained by summing the values of an index for consecutive months where it remains below zero. The period in which the value of the index remains below zero consecutively is defined as drought duration (D) and the ratio of drought severity to drought duration is defined as magnitude (M)^46^. Researchers have used this method for years^47–49^^.^

In this section, the severity, magnitude and duration values of the arid periods (where the index remained below zero) were calculated for the entire time series of SPEI-12 and SPI-12 indices calculated for each station, and the value in which each of them is the most extreme was selected. These results determined based on the station were presented with the table below. The parameters of severity and magnitude were mapped by being made areal.

**Figure 5:**
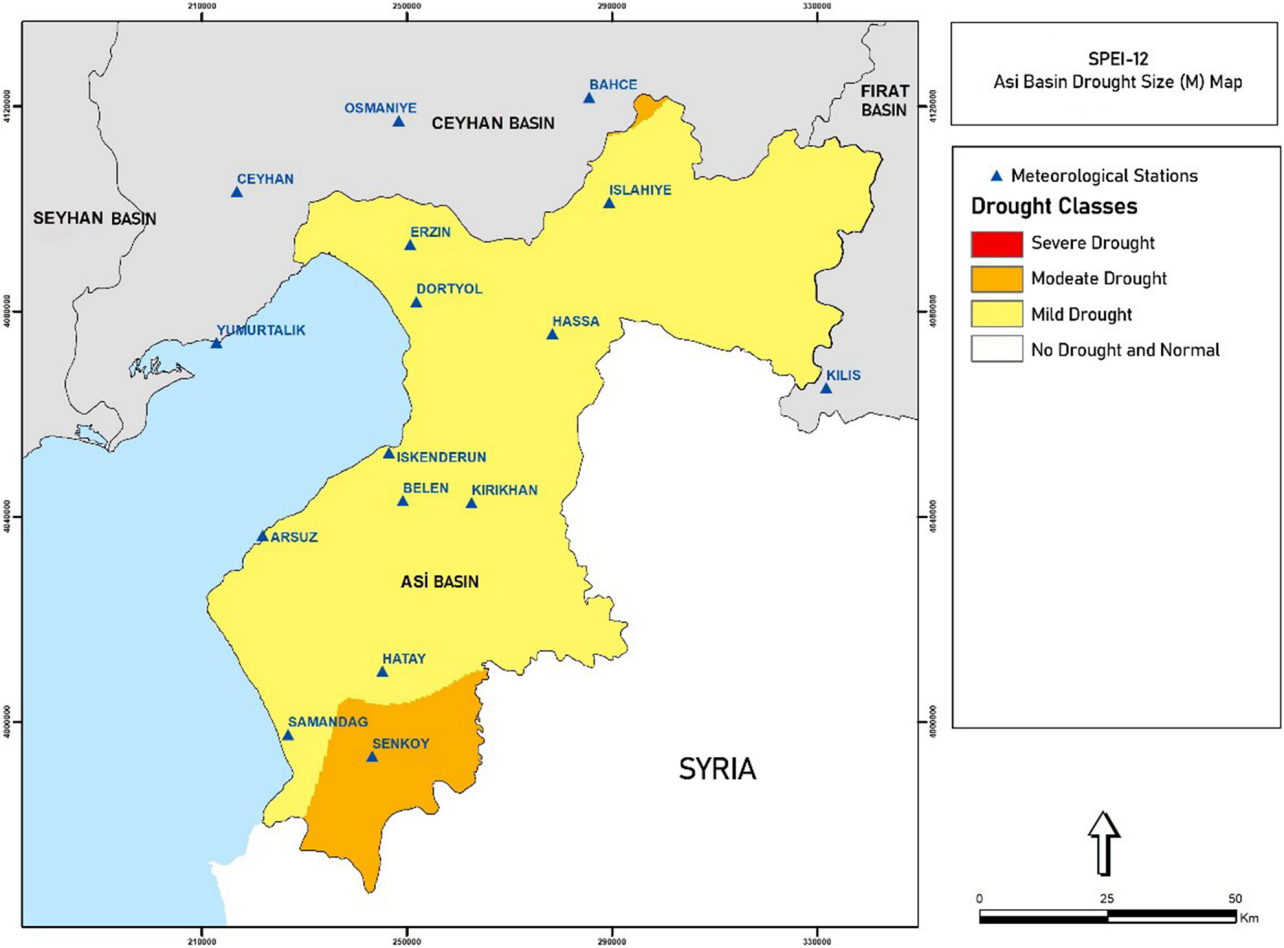
SPEI-12 drought magnitude (M) map of the Asi Basin (Global Mapper v19 and http://www.bluemarblegeo.com).

For the Standardized Precipitation Evapotranspiration Index (SPEI), the SPEI-12 drought magnitude (M) map of the Asi Basin is given in Fig. 5 as a sample. It was generated via Global Mapper program. Syria is not included to basin.

**Figure 6:**
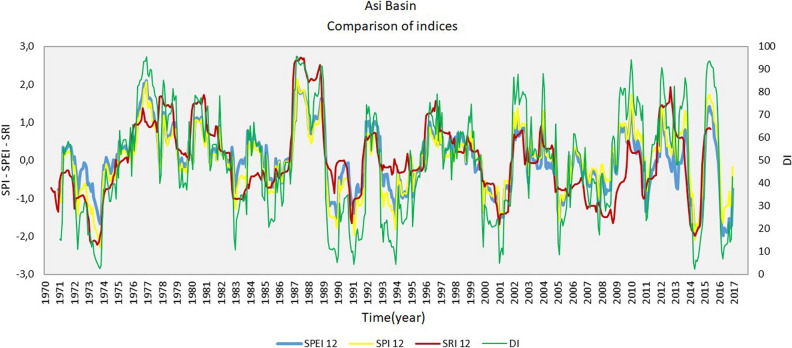
Comparison of the SPEI12, SPI12, SRI12 and DI indices for the Asi Basin .

For the Standardized Precipitation Index (SPI), the SPI-12 drought magnitude (M) map of the Asi Basin is obtained.

Drought severity (S), duration (D), and magnitude (M) were found according to the SPEI-12 and SPI-12 indices of the Asi Basin stations. According to these results:

- According to the SPEI-12 index, the station with the most drought severity was Iskenderun with − 60.7 and the station with the least drought severity was Erzin with − 23.5. In terms of duration, while the drought was effective in Hassa as the longest with 57 months, it was effective in Dörtyol and Erzin as the shortest with 31 months. In terms of the magnitude of the drought, only Şenköy fell into moderate drought category, while all other stations fell into the category of mild drought.
- According to the SPI-12 index, the station with the most drought severity was Arsuz with − 64.0 and the station with the least drought severity was Erzin with − 24.4. In terms of duration, while the drought was effective in İskenderun as the longest with 59 months, it was effective in Erzin as the shortest with 26 months. In terms of the magnitude of the drought, whereas Belen, Hatay, and Şenköy fell into moderate drought category, all other stations fell into the category of mild drought.

#### Drought risk maps

In this section, it is aimed to present the distribution maps of the drought-severity incidence percentages created using the calculated meteorological indices. Station-based index values for the SPEI-12 and SPI-12 indices were mapped by being made areal in order for them to represent periods of meteorological, hydrological and agricultural drought.

Standardized Precipitation Evapotranspiration Index (SPEI): The drought risk maps of 12-month SPEI classifications were prepared for four different severity levels (Severe Arid, Moderate Arid, Mild Arid and Normal and above). The incidence percentages of mild drought, moderate drought and severe drought are used in calculations.

Standardized Precipitation Index (SPI): In the same way, the drought risk maps of 12-month SPI classifications were prepared for four different severity levels (Severe Arid, Moderate Arid, Mild Arid, and Normal and above).

#### Drought recurrence-severity maps

Standardized Precipitation Evapotranspiration Index (SPEI): The SPEI-12 map of the 50-year Return Period for the Asi Basin is given in obtained.

Standardized Precipitation Index (SPI): The SPI-12 map of the 50-year Return Period for the Asi Basin is obtained.

As a result of the conducted analyses, the values of SPEI-1, SPEI-6, SPEI-12, SPEI-24, SPI-1, SPI-6, SPI-12, SPI-24 indices which might recur according to 5, 10 and 50-year Return Periods in 50% of the basin were colored according to the drought categories they represented and they are given in Table 1.

**Table 1:**
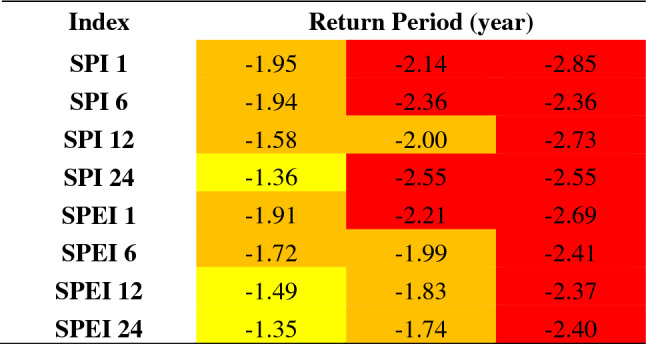
Index values that may repeat every 5, 10 and 50 years in 50% of the basin area.

According to the results given in Table 1:

- It is expected that once every 5 years, 50% of the basin will experience mild drought according to SPI-24, SPEI-12, SPEI-24 indices and moderate drought according to SPI-1, SPI-6, SPI-12, SPEI-1, SPEI-6 indices.
- It is expected that once every 10 years, 50% of the basin will experience moderate drought according to SPI-12, SPEI-6, SPEI-12, and SPEI-24 indices and severe drought according to SPI-1, SPI-6, SPI-24, and SPEI-1 indices.
- It is expected according to all indices that once every 50 years, 50% of the basin may experience severe drought.
- As a result of examining all the indices together, the periods in which the indices jointly pointed out drought for the Asi Basin were determined as the years 1973–1974, 1989–1991, 1993–1994, 2000–2001, 2004–2005, 2014 and 2016 is given as in Fig.  6.

## Discussion

Within the scope of the study, first, appropriate meteorological and runoff observation stations located in the basin were identified and their incomplete data were completed. Trend analyses were carried out to determine the change in the time series of the stations. The data of the stations was made areal and trend analyses were performed by creating time series on the basis of the basin and sub-basin. As a result of these analyses, it was seen that there was an upward trend in total annual precipitation across the Asi Basin; however, this finding was not significant at a 95% confidence level. An obvious upward trend was seen in average annual temperatures both sub-basin and basin-based. Due to the insufficient number of stations measuring evaporation, an areal evaluation could not be made. Whereas an increase was determined in some stations in the station-based evaluation, a decrease was determined in some other stations. As a result of the trend analysis conducted on the Asi Basin runoff series, it was determined that there was a downward trend in runoffs; however, this trend was not significant at the 95% confidence level.

In the context of the study, agricultural, hydrological and meteorological drought analyses were conducted using available data for the 47-year time period between 1970 and 2016. A total of 25 indices were used, including Standardized Precipitation Index (SPI1, SPI3, SPI6, SPI9, SPI12, SPI18, SPI24, SPI48), Standardized Precipitation Evapotranspiration Index (SPEI1, SPEI3, SPEI6, SPEI9, SPEI12, SPEI18, SPEI24, SPEI48), and Standardized Runoff Index (SRI1, SRI3, SRI6, SRI9, SRI12, SRI12, SRI18, SRI24, SRI48).

For each index, the threshold values corresponding to normal and above, mild arid, moderate arid, and severe arid classes were determined and all analyses were conducted by taking these values into account. Risk analysis was performed by determining the incidence percentages corresponding to the drought classes for all indices. Risk analysis was conducted both station and basin-based. The results were presented by visualizing through maps and graphs.

It was observed that the indices calculated within the scope of the study had high relations with each other. It was determined that indices calculated for the same periods showed higher correlation than those calculated for different periods.

While selecting drought indices; The length, reliability and basin specificity of the data series were taken into consideration. SPI and SPEI are used to determine meteorological, agricultural and hydrological drought, DI is used only for meteorological drought, SRI is only used to calculate hydrological drought in the literature.

The indices mostly used in drought analysis are based on precipitation and evaporation values. It is controlled by temporal variability. The effect of temperature on drought is important. To manage the drought that will be affected by climate change, it is necessary to know the trend of the drought. Although all indices show high correlation, it is appropriate to choose the most suitable index for the basin as SPEI. It is thought to be an index group that will be recommended for other basins with similar hydrographic and morphological features.

Despite the decrease in precipitation, it can be said that the agricultural activities in the region have reduced the effect of drought. However; drought management plans should be made for the basin in the Mediterranean region, which will be most affected by climate change, salinification, oceanic forcing and necessary measures should be taken.

## Method

### Drought analysis methods

#### Deciles index (DI)

**Figure 7:**
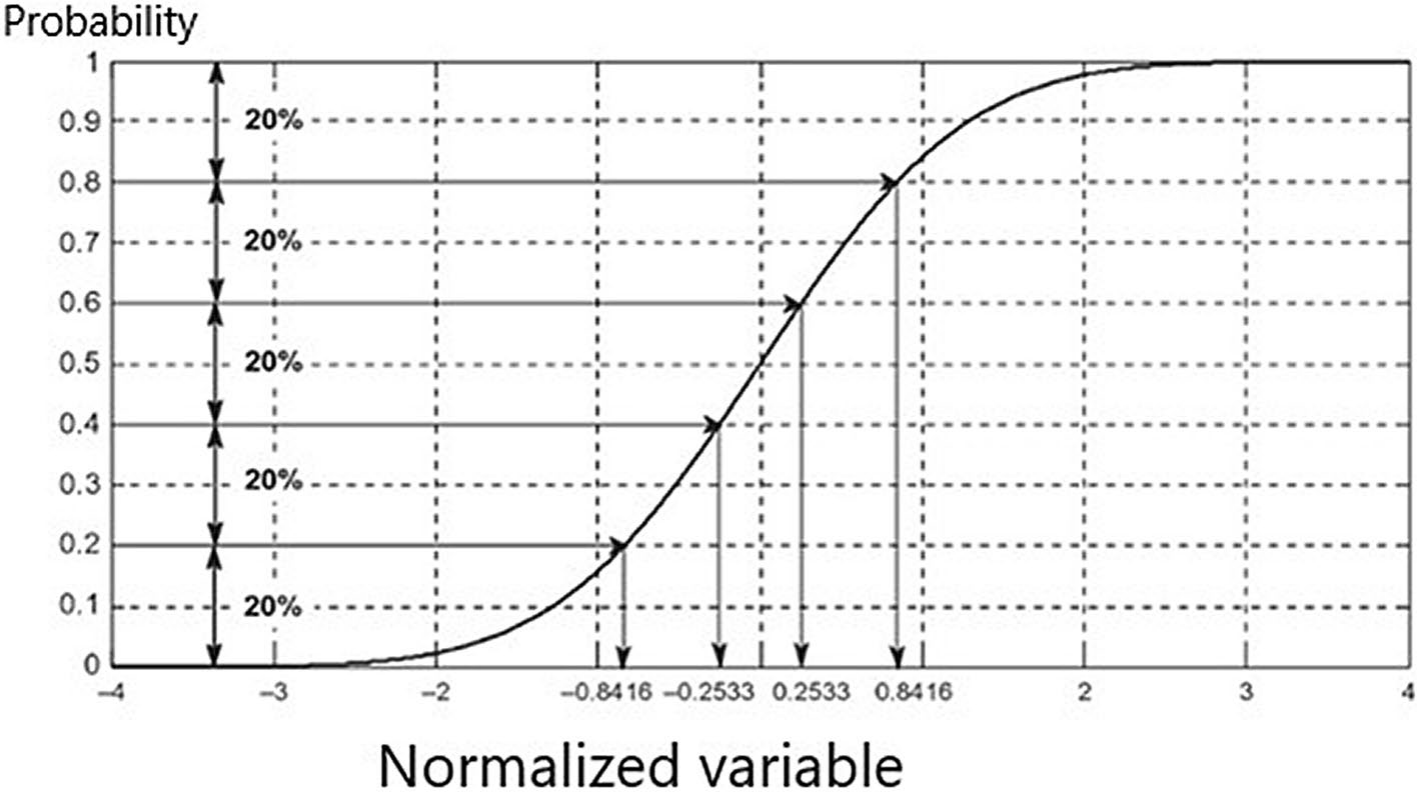
Five groups created for a standard normally distributed variable.

The deciles index has a simple mathematical approach proposed by Gibbs and Maheri^50^ for the analysis of droughts in Australia. The only data required to calculate it is precipitation data and does not require any assumptions. In this method, long precipitation series are grouped by being divided into deciles and the deciles, in which the precipitation data obtained as daily, weekly, monthly or yearly takes place, determines the degree of drought severity^51^. To calculate the deciles index, first, the empirical cumulative probability distribution was obtained by ranking the precipitation data in ascending order for the designated meteorological stations in the selected period. Then, the DI was calculated by dividing the cumulative probabilities into equal sub-ranges. The obtained groups are given in Fig. 7^51,52^.

- Deciles 1–2 (the lowest 20%): Much below the normal
- Deciles 3–4 (next low 20%): Below the normal
- Deciles 5–6 (middle 20%): Near the normal
- Deciles 7–8 (next high 20%): Above the normal
- Deciles 9–10 (the lowest 20%): Much above the normal

In the DI approach, it is considered that the probability of experiencing much lower precipitation than normal is no more than 20%^53^. Drought intensity and threshold values used in calculations made according to DI Index are given in Table 2.

**Table 2:** DI Drought classification and threshold values.

Threshold values	Drought classes
> % 75	Normal and above
% 75–% 65	Mild drought
% 65–% 55	Moderate drought
< %55	Severe drought

#### Standardized precipitation index (SPI)

The SPI, which is the most widely used meteorological drought index, is an index that bases on precipitation for different time periods and ignores other effects^54^. While negative SPI values indicate the lack of precipitation, positive SPI values indicate the precipitation excess. The intensity of the drought event can be classified according to the magnitude of negative SPI values. SPI drought classes are derived from precipitation series with the normal distribution. However, the probability distribution function of the precipitation series generally matches the Gamma distribution rather than the normal distribution. Therefore, when calculating SPI, the probabilities obtained from the probability function of the Gamma distribution are normalized using the inverse function of the normal distribution, and precipitation series are created. Since this standardization is done, the mean of the index is zero and its variance is one. SPI is calculated as follows by dividing the difference of precipitation from the average for a given timescale by the standard deviation of the series:

1 $$SPI=\frac{{x}_{i}-{x}_{j}}{\sigma }$$

In this equation, ${x}_{i}$ refers to the current precipitation in the studied period, ${x}_{j}$ refers to the mean precipitation of the series, and $\sigma$ refers to the standard deviation of the series. With SPI, it is possible to express the lack of precipitation on multiple timescales. Lack of precipitation at different time scales can be effective on different water sources. For example, soil moisture may be affected by the lack of precipitation in a shorter time, while depots may be affected in longer periods. Therefore, SPI can be calculated on 3, 6, 9, 12, 24, and 48-month time scales^55^. SPI-3 can be used to understand short-and medium-term humidity conditions, SPI-6 medium-term precipitation trends, SPI-9 medium-term precipitation patterns, and SPI-12 long-term precipitation patterns^50^. Different systems and regions can be affected by drought conditions at different time scales. For example, whereas changes in groundwater, river flow, and reservoir volume can react to precipitation anomalies that occur in the relatively long term, soil moisture changes can show variances considerably due to relatively short-term precipitation anomalies. The fact that SPI can be calculated for different time intervals has enabled this index to be applied in different areas. Therefore, SPI may ensure detection of different types of drought. SPI is calculated for a specific location and desired duration based on long-term precipitation observations. The appropriate probability distribution matching these long-term observations is determined and converted to the normal distribution in a way that the mean SPI for that location and the desired time is “0” and the standard deviation is “1”. In this study, the gamma distribution, which is the probability distribution best suited to climatic precipitation series ^51^ and which is generally preferred in drought literature^53^, was used. When calculating the SPI, the following stages were followed^25^: 1. Conversion of the probability distribution function of raw precipitation data to gamma probability distribution function. 2. For the precipitation probabilities obtained from the Gamma probability distribution function, calculation of standardized precipitation series (i.e. SPI values) by using the inverse-standard normal distribution function. SPI may represent wet periods, but it is often used to assess the duration and severity of drought events. When SPI values are consistently negative and its lowest value is below − 1, it refers to arid periods; and it is assumed to continue in this negative period until it receives 0 value. Thus, each arid period is defined with a severity value for the start, end, and each month in which the drought continues. Drought severity is the absolute value of the sum of SPI values of each month in which the drought event occurs. The drought severities and threshold values proposed by McKee ^20^ for SPI and used widely are given as in Table 3.

**Table 3:** SPI drought classification and threshold values.

Threshold values	Drought severity
2 and above	Extremely humid
1.5–1.99	Very humid
1–1.49	Moderately humid
0.5–0.99	Near normal (very little humidity)
− **0.499 to 0.499**	**Normal**
− 0.5 to − 0.99	Near normal (very little arid)
− 1 to − 1.49	Moderately arid
− 1.5 to − 1.99	Severely arid
< − 2	Extremely arid

#### Standardized precipitation evapotranspiration index (SPEI)

The Standardized Precipitation Evapotranspiration Index (SPEI) is a more recent drought index than other indices^56^. SPI is based on two basic assumptions:

- The importance of precipitation is far greater than any other variable that can affect the severity of the drought.
- Drought is controlled only by temporal variability, which occurs in precipitation. In calculating SPEI, the effect of temperature on drought is also considered and this makes it different and superior to SPI. For this reason, SPEI is an ideal index for studying climate change with climate model projections.

SPEI is an index based on precipitation and potential sweating and evaporation values. Thus, SPEI can take into account changes in evaporation values that are based on temperature change. The SPEI calculation requires complete time series data for both precipitation and potential evapotranspiration (temperature when using the Thornwaite method). Because of this, it is not possible to calculate SPEI for places where there is insufficient data. The longer the data, the more robust the results that the calculations produce^31^. SPEI takes into account cumulative climatic water budget (precipitation—potential sweating and evaporation) anomalies, and the SPEI calculation covers the conversion of long-term observations such as SPI to a normal distribution by detecting the appropriate probability distribution^57^. However, the probability distribution used in SPEI is a 2-parameter log-logistic distribution instead of a 2-parameter gamma distribution^58^. The first stage in the SPEI calculation is finding of the potential sweating and evaporation (i.e. evapotranspiration); however, the calculation of potential sweating and evaporation is quite difficult. There are several methods in the literature for calculating potential sweating and evaporation. In this study, Thornthwaite method was used to calculate the required potential sweating and evaporation values in SPEI calculations because this method only required monthly average temperature data^56^. In the second stage, in the context of the equation given below, a simple criterion for water excess or water scarcity (*D*_*i*_) is obtained by subtracting the potential sweating and evaporation value (*PET*_*i*_) calculated by using the Thornthwaite method from the precipitation value for the month studied:

2 $${\mathrm{D}}_{i}={\mathrm{P}}_{i}-\mathrm{ PE}{\mathrm{T}}_{i}$$

In the third stage, *D*_*i*_ values are converted to log-logistic probability distribution function. In the fourth and final phase, *D*_*i*_ series (that is, SPEI values), which have been standardized like SPI by using the inverse-standard normal distribution function for water excess or lack probabilities obtained from the log-logistic probability distribution function, are obtained. The fact that SPEI is 0 indicates a value equivalent to 50% of the cumulative probability of water excess or water scarcity (*D*_*i*_) according to the log-logistic distribution. In many subjects, such as computational methods and detection and monitoring of arid periods, SPEI resembles SPI. Like SPI, SPEI also has a severity scale where both positive and negative values are calculated. In this way, it is able to ensure the detection of both rainy and dry periods. Similarly, since SPEI values are normalized, it is an index that can be applied and compared in all climate regimes. SPEI can be calculated monthly for time intervals from 1 to 48 months or more.

#### Standardized runoff index (SRI)

SRI is a drought index developed in 2008 as an expression of hydrological drought and based on SPI methodology. Unlike SPI, runoff data is used in SRI calculation. SRI is calculated as follows by dividing the difference of runoff values from the mean value for a given timescale by the standard deviation of the series:

3 $$SRI=\frac{{x}_{i}-{x}_{j}}{\sigma }$$

where ${x}_{i}$ refers to the current runoff in the studied period, ${x}_{j}$ refers to the mean of the series, and $\sigma$ refers to the standard deviation of the series. SRI is an easy-to-calculate index, such as SPI, since it requires only the use of runoff data. Like SPI, SRI can also be calculated on a daily or monthly basis using both observed and predicted runoff data. Thus, it can provide information about high and low runoff periods related to floods and drought. Thanks to SRI, hydrological requirements of a location can be tracked on multiple time scales ^7^. SRI results can be evaluated by comparing them with SPI analysis of the same region. Studies conducted in terms of identifying the relationship between precipitation and runoff have shown a strong relationship between SPI and SRI^58^. When calculating SRI, it is important that the stations represent the basin and that the runoff series are natural. In analyses of droughts, when hydrological droughts are being studied, 9–12-month SRI results are preferred because they reflect a full hydrological precipitation period. The drought severities and threshold values commonly used in the literature for SRI are the same values. Because the calculation method of SRI is the same as the calculation methods of SPI and SPEI, the same threshold values were used.

#### Drought classification and threshold values used for the drought analysis of the Asi Basin

In the context of the Asi Basin drought analysis studies, instead of drought severity and threshold values methods whose methods are explained in this section and that are preferred in the literature, four main classes of drought severity were used to allow all indices to be compared consistently. These were designated as Severe Drought, Moderate Drought, Mild Drought, and No Drought-Normal/Humid State. Table 4 shows the drought classification and threshold values of the indices used in the Asi Basin drought analysis study.

**Table 4:**
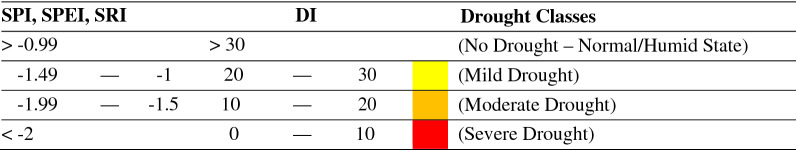
Drought classification and threshold values used for the Asi Basin drought analysis.
